# Expression of the *glioma-associated oncogene homolog (GLI) 1 *in human breast cancer is associated with unfavourable overall survival

**DOI:** 10.1186/1471-2407-9-298

**Published:** 2009-08-25

**Authors:** Anette ten Haaf, Nuran Bektas, Sonja von Serenyi, Inge Losen, Elfriede Christel Arweiler, Arndt Hartmann, Ruth Knüchel, Edgar Dahl

**Affiliations:** 1 Molecular Oncology Group, Institute of Pathology, University Hospital of the RWTH Aachen, Pauwelsstrasse 30, 52074 Aachen, Germany; 2 Institute of Pathology, University Hospital Bonn, Sigmund-Freud-Strasse 25, 53127 Bonn, Germany; 3 Institute of Medical Statistics, University Hospital of the RWTH Aachen, Pauwelsstrasse 30, 52074 Aachen, Germany; 4 Institute of Pathology, University Hospital Erlangen, Krankenhausstrasse 12, 91054 Erlangen, Germany

## Abstract

**Background:**

The transcription factor GLI1, a member of the GLI subfamily of Krüppel-like zinc finger proteins is involved in signal transduction within the hedgehog pathway. Aberrant hedgehog signalling has been implicated in the development of different human tumour entities such as colon and lung cancer and increased GLI1 expression has been found in these tumour entities as well. In this study we questioned whether GLI1 expression might also be important in human breast cancer development. Furthermore we correlated GLI1 expression with histopathological and clinical data to evaluate whether GLI1 could represent a new prognostic marker in breast cancer treatment.

**Methods:**

Applying semiquantitative realtime PCR analysis and immunohistochemistry (IHC) GLI1 expression was analysed in human invasive breast carcinomas (*n *= 229) in comparison to normal human breast tissues (*n *= 58). *GLI1 *mRNA expression was furthermore analysed in a set of normal (*n *= 3) and tumourous breast cell lines (*n *= 8). IHC data were statistically interpreted using SPSS version 14.0.

**Results:**

Initial analysis of GLI1 mRNA expression in a small cohort of (*n *= 5) human matched normal and tumourous breast tissues showed first tendency towards GLI1 overexpression in human breast cancers. However only a small sample number was included into these analyses and values for GLI1 overexpression were statistically not significant (*P *= 0.251, two-tailed Mann-Whitney U-test). On protein level, nuclear GLI1 expression in breast cancer cells was clearly more abundant than in normal breast epithelial cells (*P *= 0.008, two-tailed Mann-Whitney U-test) and increased expression of GLI1 protein in breast tumours significantly correlated with unfavourable overall survival (*P *= 0.019), but also with higher tumour stage (*P *< 0.001) and an increased number of tumour-positive axillar lymph nodes (*P *= 0.027). Interestingly, a highly significant correlation was found between GLI1 expression and the expression of SHH, a central upstream molecule of the hedgehog pathway that was previously analysed on the same tissue microarray.

**Conclusion:**

Our study presents a systematic expression analysis of GLI1 in human breast cancer. Elevated levels of GLI1 protein in human breast cancer are associated with unfavourable prognosis and progressive stages of disease. Thus GLI1 protein expression measured e.g. by an IHC based scoring system might have an implication in future multi-marker panels for human breast cancer prognosis or molecular sub typing. The highly significant correlation between SHH and GLI1 expression characterises GLI1 as a potential functional downstream target of the hedgehog signalling pathway in human breast cancer as well. Furthermore, our study indicates that altered hedgehog signalling may represent a key disease pathway in the progression of human breast cancer.

## Background

The hedgehog (Hh) signalling pathway is known to be essential for multiple aspects of embryonic development. It is implicated in processes of organ patterning, cell differentiation and cell proliferation but it also plays a crucial role in the development of the limb, lung, brain and foregut [1]. An active involvement of Hh signalling has been shown for a variety of human tumour entities including tumours of skin, cerebellum, muscle, lung, digestive tract, pancreas and prostate [2-7]. Furthermore, the malignancy of tumours and their progression to metastatic stages have been associated with the activity of the Hh signalling pathway [8,9]. GLI1 is a member of this pathway and belongs to the family of GLI transcription factors including GLI1, GLI2 and GLI3. These transcription factors act as downstream mediators of Hh signalling and they share in common a DNA binding zinc finger domain [10]. The human *GLI1 *gene is located on chromosome 12q13.2-q13.3 [11] and functions as an activator of transcription [12]. Downstream targets of GLI1 signalling include molecules with regulatory effects on cell cycle and apoptosis such as Cyclin D2 or FOXM1 in basal cell carcinomas [13,14]. Several studies on GLI1 analysed potential involvement of this transcription factor in tumour developmental processes. Dahmane et al. (1997) [15] showed that ectopic expression of GLI1 in the basal cells of the embryonic frog epidermis is able to induce basal cell carcinomas. Additionally in human oesophageal squamous cell carcinomas an association was found between the expression of GLI1 and depth of tumour invasion, status of lymph node metastasis, as well as unfavourable overall survival [16].

SHH is another important member of the Hh signalling cascade which acts upstream of GLI1 in the activation process of the Hh pathway [17]. It binds to the receptor Patched (PTCH) 1 or 2, which then relieves repression of the membrane protein Smoothened (SMO), a G protein-coupled receptor related protein [9,17]. The relief of SMO inhibition leads to an activation of GLI1 [18]. As a result of this GLI1 accumulates in the nucleus [19] where it controls the expression of typical Hh target genes [20]. Thus SHH expression is assumed to have a direct impact on GLI1 activity. A significant correlation between increased expression of both SHH and GLI1 in human breast cancer would support the hypothesis that aberrant Hh signalling contributes to breast cancer development or progression. Therefore we performed a systematic expression analysis of GLI1 in human breast cancer on the mRNA and protein level and correlated GLI1 expression with clinicopathological patient characteristics. Subsequently we compared GLI1 expression data to previous data on the expression of SHH in the same collective of breast tumours.

## Methods

### Patient samples and breast cancer tissue microarray (TMA)

Samples of tumourous and normal breast tissues were derived from patients that underwent primary surgery for breast cancer at the Departments of Gynaecology at the University Hospitals of Aachen and Regensburg. All patients gave informed consent for retention and analysis of their tissue for research purposes and the Institutional Review Board of the participating centre approved the study.

Tissues were immediately snap-frozen in liquid nitrogen. To determine the percentage of tumour cells H&E staining was carried out on sections of each tissue. Only samples that contained more than 70% tumour cells were selected for further analysis. In order to determine the precise ratio of cancer cells the dissected tumours were macroscopically and microscopically (high power field; ×400) examined by a team of experienced pathologists. Clinicopathological characteristics of the tumours are listed in Table 1. GLI1 expression was further analysed by immunohistochemistry on a breast cancer TMA containing 204 breast cancer specimens and 46 normal breast tissue specimens. This TMA has previously been described in detail [21]. In short, tissue cores on the TMA were derived from non-selected, formalin-fixed and paraffin-embedded primary breast cancer specimens that had been diagnosed at the Institute of Pathology, University of Regensburg, Germany between 1994 and 2002. Prior to construction of the TMA haematoxylin and eosin (H&E) staining was carried out to identify representative tumour areas. Slides for all specimens were evaluated with help of an experienced surgical pathologist (A.H.) and tumour grades were predicted on basis of the Elston and Ellis grading definitions [22]. Amplification of the *HER2 *gene in tumours with a medium HER2 staining intensity of "score 2" was tested by additional FISH analyses in order to determine HER2 amplification of these tumours. As 62% of the analysed tumours were negative for *HER2 *gene amplification they were included into the group of HER "weak expressers" (0–2+ (weak) vs. 3+ (strong)). Tissues on the TMA were derived from patients with a median age of 56 years (range from 25 to 82 years). All clinical follow-up data were derived from the Central Tumour Registry, Regensburg, Germany with a median follow-up period of 78 months (range 0–148 months). Both studies were approved by the Institutional Review Board of each participating centre and all patients agreed to retention and analysis of their tissues for research purposes.

**Table 1:** Clinicopathological characteristics of primary breast carcinomas (n = 5)

Tumour Nr.	Histological type	**Tumour stage**^a^ (pT)	Lymph node status (pN)	Histological grade (G)
T1	ductal	pT1	pN0	G3
T2	ductal	pT2	pN1	G3
T3	ductal	pT1	pN1	G3
T4	ductal	pT2	pN1	G3
T5	ductal	pT3	pN3	G2

^a^According to UICC: TNM Classification of Malignant Tumours. 6^th ^edn (2002) Sobin LH, Wittekind CH (eds) Wiley: New York

### Cell lines

The human mammary epithelial cell lines HMEC, MCF12A and MCF10A as well as the breast cancer cell lines T47D, MCF7, ZR75-1, SKBR3, MDA-MB468, BT20, MDA-MB231 and MDA-MB435s were obtained from the ATCC (Rockville, MD, USA) and cultured as previously described [23].

### DNA and RNA extraction and reverse transcription

For DNA extractions tissue samples were lysed overnight at a temperature of 56°C with 800 rpm shaking. After 12 h incubation time DNA was isolated according to the instructions of the blood and cell culture DNA kit (Qiagen, Hilden, Germany). RNA was extracted following the manufacturer's protocols for Trizol isolation (Invitrogen, CA). For RNA extractions from paraffin-embedded tissues representative sections were determined, deparaffinised and conventionally rehydrated in a decreasing alcohol-series prior to extraction. Using the Reverse Transcription System (Promega Corporation, WI) cDNA was synthesised using 1 μg of each RNA.

### Semiquantitative realtime PCR

The IQ™5 realtime PCR Detection System was used. All reactions consisted of 10 μM forward primer, 10 μM reverse primer, 10 μl iQ™ SYBR^® ^Green Supermix (Bio-Rad Laboratories, Munich, Germany) and 1–2 μl of cDNA as PCR template in a final reaction volume of 20 μl. Gene expression was quantified using the comparative C_T _method, which normalises the C_T _values to an internal housekeeping gene (*GAPDH*) and calculates the relative expression values [24]. Primer sequences for *GLI1 *and *GAPDH *are listed in Table 2. The cycling conditions were set up to an initial denaturation step at 95°C for 3 min, followed by 43 cycles with denaturation at 95°C for 15 s, annealing of the primers at 65°C for 15 s and elongation at 72°C for 20 s. Melting curve analyses as well as gel electrophoretical analyses were performed to verify the specificity of the PCR products. To ensure accuracy of the results all reactions were performed in triplicate and arithmetic means were represented for each sample.

**Table 2:** Primer sequences and annealing temperatures used in this study

Sequence		Annealing temperature (°C)
***Semiquantitative Realtime PCR (Light Cycler)***		

*GLI1*	Forward: 5'- AGGGAGTGCAGCCAATACAG -3' Reverse: 5'- ATTGGCCGGAGTTGATGTAG -3'	65

*GAPDH*	Forward: 5'- GAAGGTGAAGGTCGGAGTCA -3' Reverse: 5'- AATGAAGGGGTCATTGATGG -3'	65

### GLI1 immunohistochemistry

Immunohistochemical analysis of the TMA was carried out according to the manufacturer's instructions (Advance Kit, DAKO K4068, Glostrup, Denmark). Tissues were deparaffinised and rehydrated followed by a microwave heating for 30 min in 10 mM sodium citrate buffer (pH 7.2). Peroxidase blocking solution (DAKO S 2023) was used for blocking of endogenous peroxidises. The polyclonal primary antibody GLI1 (Santa Cruz sc-20687, CA) was applied (1:50 dilution) overnight at 4°C. Sections from human basal cell carcinoma in which GLI1 expression has already been described by Ghali et al. (1999) served as positive controls. The application of primary antibodies to tissue sections was omitted in negative controls. Signals were developed in a 30 min incubation step with the Advance Kit HRP against rabbit/mouse (DakoCytomation K4065) followed by 3,3'-Diaminobenzidine (DAB) detection. Slides were counterstained with haematoxylin and after dehydration mounted in Vitro-Clud (Langenbrinck, Emmendingen, Germany). Intensity and scoring of the immunohistochemical staining was determined through an experienced pathologist (N.B.) following instructions of the scoring system suggested by Remmele and Stegner [25].

The Immuno-Reactive-Score (IRS) is product of staining intensity (graded between negative = 0 and strong = 3) and the percentage of positively stained cells (graded between 0 and 4, being 1 = <25%, 2 = 25–50%; 3 = 51–75%, and 4 = >75%, respectively). Cases with discrepancies in IRS score were discussed together with other pathologists until consensus was reached.

### Statistics

Immunohistochemical data were statistically analysed using the SPSS software version 14.0 (SPSS GmbH Software, Munich, Germany). Differences with a p-value < 0.05 were defined to be statistically significant. A non-parametrically two-tailed Mann-Whitney U-test was used for determination of differences in the GLI1 expression levels. Additionally a two-sided Fisher's exact test was performed to analyse possible associations between GLI1 expression and clinicopathological parameters. Logistic regression analysis included all parameters that were significantly prognostic in univariate analysis at a level of *P *< 0.2. Earlier studies from our group analysed the protein expression of Hh member SHH on the same set of tissues specimens. The results from these analyses were used in a second Fisher's exact test to compare the levels of protein expression for the two Hh members SHH and GLI1 in normal breast and tumourous breast tissues. Overall survival (OS) and Recurrence-free (RFS) were calculated in accordance with the Kaplan-Meier equation.

## Results

### GLI1 mRNA expression in cancerous human epithelial breast cell lines

**Figure 1:**
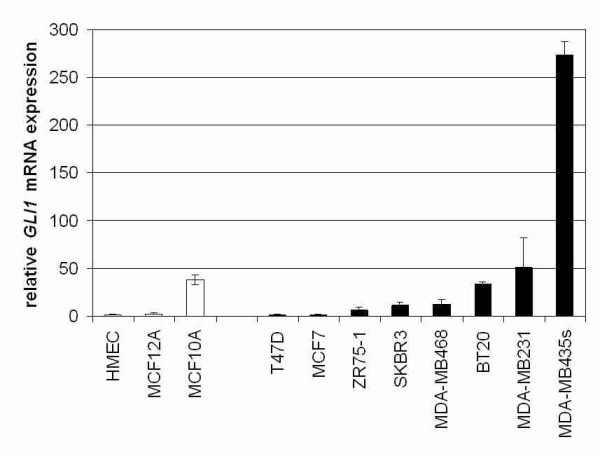
**Elevated expression of *GLI1 *mRNA in breast cancer cell lines**. Semiquantitative realtime PCR (Light Cycler) analysis for *GLI1 *expression was performed on reverse transcribed RNA extracted from non-malignant (grey bars) and malignant breast cell lines (black bars). Expression data were related to *GLI1 *mRNA expression in the human mammary epithelial cell line HMEC (set to 1). In five of eight analysed malignant mammary cell lines (SKBR3, MDA-MB468, BT20, MDA-MB231 and MDA-MB435s) increased levels of *GLI1 *mRNA expression were found. *GLI1 *mRNA expression in normal human epithelial mammary cell lines HMEC and MCF12A cells is relatively low, except for cell line MCF10A which also presents abundant *GLI1 *expression.

Our study was initiated by a *GLI1 *mRNA expression analysis in a set of normal human mammary epithelial cell lines in comparison to malignant human mammary cell lines by semiquantitative realtime PCR (Figure 1). Compared to the primary cell line HMEC (human mammary epithelial cells) and the immortalised benign cell line MCF12A, *GLI1 *expression was clearly upregulated in the analysed set of breast cancer cell lines. However, the immortalised benign cell line MCF10A also demonstrates abundant *GLI1 *expression. Differences were statistically not significant (*P *= 0.414, two-tailed Mann-Whitney U-test).

### GLI1 mRNA expression in primary breast cancers

**Figure 2:**
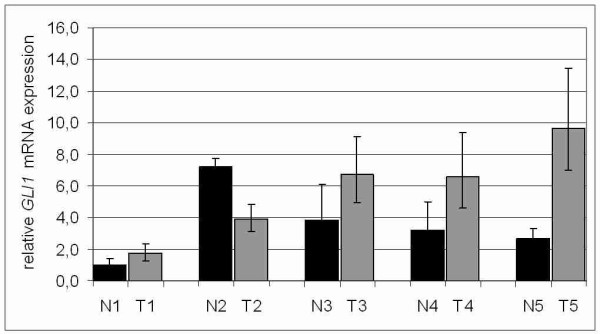
***GLI1 *expression in primary breast cancer**. Realtime PCR analysis of *GLI1 *mRNA expression in a set of five matched pairs (N = normal *vs*. T = tumour) revealed increased levels of *GLI1 *expression in tumourous tissue for four of the five analysed matched pairs. Expression level of each sample is normalised to its GAPDH expression and related to normal breast tissue (N1) (set to 1).

Next we analysed *GLI1 *mRNA expression in a set of matched tumour and normal breast tissue samples (*n *= 5; Figure 2). Four of five analysed matched samples exhibited increased levels of *GLI1 *mRNA expression in the tumourous breast tissues compared to their adjacent normal tissues. However overexpression of GLI1 in the analysed breast tumours was statistically not significant (*P *= 0.251, two-tailed Mann-Whitney U-test).

### GLI1 protein expression analysed on the breast cancer TMA

**Figure 3:**
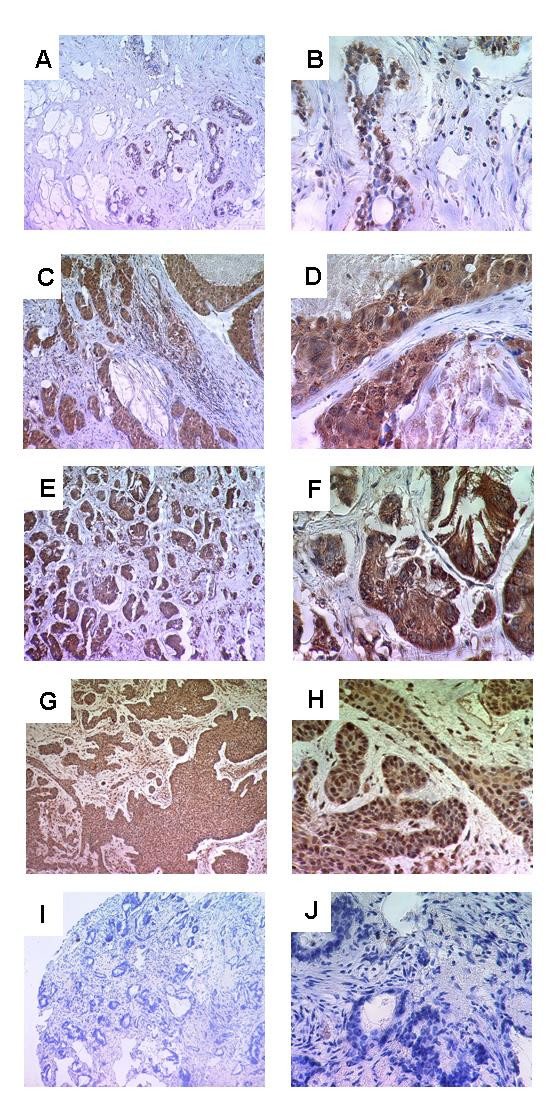
**Immunohistochemical expression analysis of GLI1 protein in normal breast tissue, non-invasive and invasive breast tumours using a TMA**. (A, B) In normal breast tissue low levels of GLI1 expression are detectable in the cytoplasm (median IRS = 4) and in the nucleus (median IRS = 6). In ductal carcinomas *in situ *of high grade type (C, D) as well as in invasive breast carcinomas (E, F) nuclear GLI1 expression is more intense than in normal breast tissues (median IRS = 8), whereas the cytoplasmic GLI1 expression is weak (median IRS = 3). (G, H) Representative tissue section from human basal cell carcinoma which served as a positive control for GLI1 staining. (I, J) Negative control – benign breast tissue. Magnifications: A, C, E, G, I: 100×; B, D, F, H, J: 400×.

Next, GLI1 protein expression and localisation was studied on a large TMA including normal (*n *= 46) and tumourous breast tissues (*n *= 204). Immunohistochemical stainings were processed using a primary GLI1 antibody (Santa Cruz sc-20687) that has been verified for specificity as described previously [26,27]. GLI1 immunohistochemical staining was detectable in both the cytoplasm and the nucleus of benign and malignant mammary epithelial cells. However, nuclear expression was more abundant (Figure 3), and as GLI1 is a transcription factor, nuclear GLI1 expression was scored for subsequent data analyses and correlation studies. Considering the whole TMA 97% (198/204) of the analysed breast carcinomas and 76% (35/46) of all analysed normal breast tissues were shown to present abundant nuclear GLI1 protein expression. In the nucleus of normal breast tissues, GLI1 protein expression was less abundant (median IRS = 6) than in the nucleus of breast carcinoma cells (median IRS = 8) (*P *= 0.008, two-tailed Mann-Whitney U-test). Intensities of nuclear GLI1 protein varied strongly between the different breast tumours as IRS values detected on the TMA ranged between 2 and 12 (3.1% and 24.6% of all analysed tumours, respectively).

Figure 3 demonstrates nuclear GLI1 expression in normal breast tissues (A and B), ductal carcinomas *in situ *of high grade type (C and D) and in invasive breast carcinomas of ductal type (E and F). Ductal carcinomas *in situ *and invasive breast tumours are both characterised by abundant nuclear GLI1 expression.

### Correlation analysis including clinicopathological parameters

GLI1 acts as a transcription factor mediating Hh signalling in embryonic and adult cells. For correlation analysis of nuclear GLI1 expression and clinicopathological data we performed an easy stratification of GLI1 expression in "low expressers" (IRS score 0 to 6) and "high expressers" (IRS score 7 to 12). A significant correlation was found between increased expression of nuclear GLI1 and tumour stage (*P *< 0.001) but also between increased expression of nuclear GLI1 and the lymph node status (*P *= 0.027) of the analysed tumours (Table 3). No significant association was found between GLI1 expression and the presence of HER2 receptor (*P *= 0.484), oestrogen receptor (*P *= 0.266) or progesterone receptor (*P *= 0.847) (Table 3).

**Table 3:** Clinicopathological and immunohistochemical parameters in relation to nuclear GLI1 immunoreactivity

Variable	Categorization	**GLI1 nuclear immunoreactivity**^b ^0–6 vs 7–12			
		n analyzable	low	high	***P***^c^
***Clinicopathological data:***					
Tumour stage^a^					
	pT1	57	34	23	**<0.001**
	pT2	87	19	68	
	pT3	12	3	9	
	pT4	28	8	20	
Lymph node status					
	pN0	78	36	42	**0.027**
	pN1-3	100	30	70	
Histologic grade					
	G1	21	10	11	0.191
	G2	81	29	52	
	G3	82	25	57	
Multifocality					
	unifocal tumour	161	57	104	0.844
	multifocal tumour	24	8	16	
Histologic type					
	ductal	152	57	95	0.312
	lobular	14	3	11	
	other	17	5	2	
					
***Immunohistochemistry (IHC):***					
Oestrogen receptor status					
	negative	53	19	34	0.484
	positive	96	29	67	
Progesterone receptor status					
	negative	109	33	76	0.266
	positive	51	20	31	
HER2 status					
	weak (0–2+)	132	45	87	0.847
	strong (3+)	31	10	21	

^a^According to UICC: TNM Classification of Malignant Tumours. 6^th ^edn (2002) Sobin LH, Wittekind CH (eds) Wiley: New York

^b^GLI1 nuclear immunoreactivity: low = IRS 0–6, high = IRS 7–12

^c^Fisher's exact test (two-sided), bold face representing significant data (*P *< 0.05)

### GLI1 overexpression correlates with unfavourable patient prognosis

**Figure 4:**
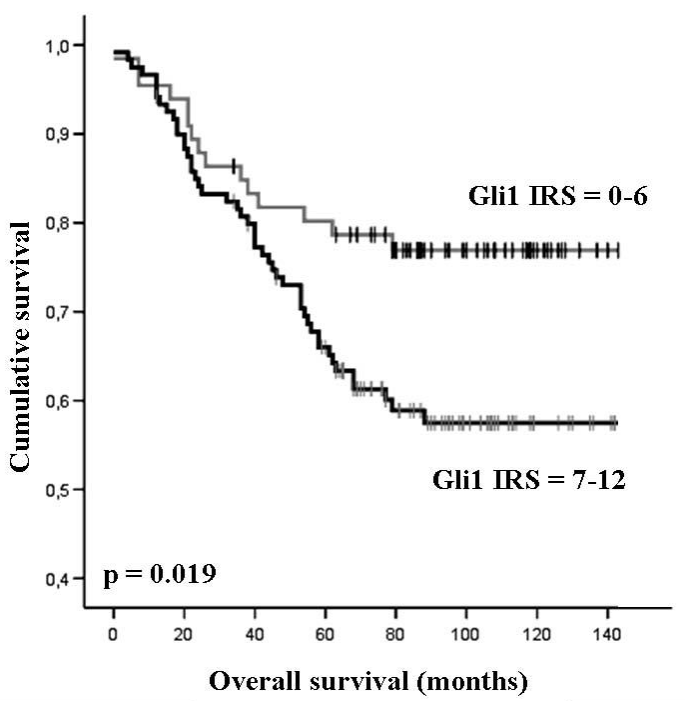
**Breast cancer patients expressing nuclear GLI1 protein show unfavourable prognosis**. Univariate Kaplan-Meier analysis was performed on the basis of GLI1 expression results derived from the TMA. Patients with low GLI1 expression (IRS = 0–6) displayed improved overall survival estimation (upper graph) compared to patients with abundant nuclear GLI1 expression (IRS 6–12; lower graph) (*P *= 0.019; univariate log-rank analysis).

To answer the question whether GLI1 overexpression might have an impact on patients' clinical outcome univariate survival probability curves were calculated based on the immunohistochemical results. Using Kaplan-Meier analysis we found that "high GLI1 expressers" (IRS 7–12) had an unfavourable overall survival prognosis (*P *= 0.019) (Table 4, Figure 4). Regarding the clinical impact of increased GLI1 protein expression on breast tumour recurrence the derived data were statistically not significant (*P *= 0.102; Table 4).

**Table 4:** Univariate analysis of factors regarding overall survival (OS) and recurrence-free survival (RFS)

Variable	Categorization	Tumour-related death (OS)			Tumour recurrence (RFS)		
		n	events	***P***^b^	n	events	***P***^b^
***Clinicopathological data:***							
Tumour stage^a^							
	low (pT 0–1)	58	10	**0.001**	56	12	**0.000**
	high (pT 2–4)	128	54		123	64	
Lymph node status							
	negative (pN 0)	78	12	**0.000**	77	14	**0.000**
	positive (pN 1–3)	101	46		98	57	
Histologic grade							
	low (G 1–2)	102	26	**0.001**	97	30	**0.000**
	high (G 3)	83	38		81	45	
Multifocality							
	unifocal tumour	162	53	0.164	157	65	0.453
	multifocal tumour	24	11		22	11	
Histologic type							
	ductal	152	51	0.614	149	65	0.562
	lobular	14	7		12	5	
	other	18	6		16	5	
							
***Immunohistochemistry (IHC):***							
Oestrogen receptor status							
	negative (IRS 0–2)	84	35	**0.006**	83	39	0.055
	positive (IRS 3–12)	66	14		63	20	
Progesterone receptor status							
	negative (IRS 0–2)	51	7	**0.000**	104	51	**0.004**
	positive (IRS 3–12)	110	48		51	12	
HER2 IHC							
	weak (0–2+)	126	48	**0.044**	133	38	**0.014**
	strong (3+)	31	17		31	15	
GLI1^c^							
	low (IRS 0–6)	66	15	**0.019**	64	23	0.102
	high (IRS 7–12)	120	49		116	53	

^a^According to UICC: TNM classification of malignant tumours. 6^th ^edn (2002) Sobin LH, Wittekind CH (eds) Wiley: New York

^b^Log rank test (two-sided), bold face representing significant data (*P *< 0.05)

^c^GLI1 nuclear immunoreactivity: low = IRS 0–6, high = IRS 7–12

### Logistic regression analysis including tumour stage (pT), lymph node status (pN) and histological grade (G)

Multivariate logistic regression analysis was carried out to evaluate the role of tumour stage, histological grade and lymph node status as indicators for GLI1 overexpression (Table 5). The odds for GLI1 overexpression (IRS 0–6 vs IRS 7–12) increased 4.9 times (95% CI 2.3 – 10.5) in patients with pT2 compared to those with pT1 (*P *< 0.001). However regression analysis including the remaining pT stages (pT2 vs pT3 and pT3 vs pT4), pN status (pN0 vs pN1-3) and tumour grade (G0 vs G1-3) failed significance as independent prognostic indicators of GLI1 overexpression.

**Table 5:** Logistic regression analysis for tumour stage, lymph node status and histological grading and GLI1 immunohistochemistry

Characteristic	*P*-value	Odds ratio	95.0% confidence interval (CI)
pT^a ^(pT1 vs pT2)	**0.000**	4.911	[2.297–10.501]
pT^a ^(pT2 vs pT3)	0.078	3.782	[0.862–16.599]
pT^a ^(pT3 vs pT4)	0.083	2.673	[0.881–8.113]
pN^a ^(pN0 vs pN1-3)	0.383	1.400	[0.658–2.979]
G (G0 vs G1-3)	0.280	1.779	[0.625–5.065]

Abbreviations: pT, tumour stage; pN, lymph node status; G, histological grading.

Significant *P*-values marked inbold face.

^a^According to UICC: TNM classification by Sobin and Wittekind (2002).

### SHH protein expression in correlation to GLI1 expression data

Previously we have analysed SHH expression on the same breast cancer TMA (ten Haaf et al. (2009), submitted). Since GLI1 is supposed to be a direct downstream target of Hh signalling in model organisms like Drosophila [28] we wanted to decipher whether there is a correlation between the SHH and GLI1 expression patterns in human breast cancer and normal human breast tissues. Indeed, we found a highly significant positive correlation between the expression data of these two Hh signalling members. (Table 6; *P *< 0.001 in both tested correlation combinations).

**Table 6:** GLI1 nuclear immunoreactivity in relation to cytoplasmic SHH reactivity; 187 human tumourous breast tissues

Variable	Categorization	**GLI1 nuclear immunoreactivity**^a^ 0–6 vs 7–12			
		n analyzable	low	high	**P**^c^
***Immunohistochemistry (IHC): 187 human tumourous breast tissues***					
SHH^b^					
	low (IRS 0–6)	88	45	43	**0.000**
	high (IRS 7–12)	99	20	79	
***Immunohistochemistry (IHC): 35 human normal breast tissues***					
SHH^b^					
	low (IRS 0–6)	23	19	4	**0.000**
	high (IRS 7–12)	12	3	9	

^a^GLI1 nuclear immunoreactivity: low = IRS 0–6, high = IRS 7–12

^b^SHH cytoplasmatic immunoreactivity: low = IRS 0–5, high = IRS 6–12

^c^Log rank test (two-sided), bold face representing significant data (*P *< 0.05)

## Discussion

GLI1, a mediator of the Hh signalling pathway has previously been implicated in the development of different human tumour entities such as oesophageal squamous cell carcinomas [16], basal cell carcinomas [29] and brain tumours [30]. Consistent with a major role in carcinogenesis highly increased levels of GLI1 expression were found in basal cell carcinomas but not in the surrounding normal breast tissues [15,29,31].

To analyse the role of GLI1 in human breast cancer we carried out an initial realtime PCR analysis and found increased levels of GLI1 mRNA expression comparing the malignant mammary cell lines SKBR3, MDA-MB468, BT20, MDA-MB231 and MDA-MB435s to a set of benign mammary cell lines (HMEC and MCF12A). *GLI1 *mRNA was especially abundant in the cell line MDA-MB435s, which is a common *in vitro *and *in vivo *model for metastatic breast cancer behaviour [32] stating a potential involvement of GLI1 signalling in human breast tumour metastasis. Interestingly we also detected high levels of GLI1 mRNA expression in the benign breast cell line MCF10A.

It is known that GLI proteins themselves are mediators of more than just the Hh signalling pathway [12] and overexpression of GLI1 is reported from several human tumour entities [16,29,30] as well as benign tissues of organs like prostate or colon [33,34]. Therefore a functional involvement of GLI1 activity in human tumour development [35] but also within cellular processes and maintenance of normal adult tissues might be concluded [33]. mRNA expression analysis was further applied to a set of five matched normal and tumourous breast tissues. The results from these analyses underlined a potential involvement of GLI1 as a transcriptional regulator important for breast cancer development as increased levels of GLI1 expression were shown in four out of five analysed samples. However data derived from these studies were statistically not significant maybe because of the small sample numbers. Therefore accuracy of this first explorative approach should be tested in a larger validation set.

GLI1 expression was also studied on protein level using a larger cohort of human breast cancers (*n *= 204) as well as normal breast tissues (*n *= 46). We were able to show significantly increased levels of nuclear GLI1 protein expression (median IRS = 8) in human breast carcinomas in comparison to normal breast tissues (median IRS = 6). Furthermore intensities of GLI1 expression varied strongly between the different analysed tissues. These data are in accordance to earlier findings from Kubo et al. (2004) where nuclear GLI1 overexpression was found in a set of human breast cancers (*n *= 52) and ratios of GLI1 expressing carcinoma cells varied strongly between the different breast tumour samples [35]. Thus overexpression of GLI1 may not be a general characteristic of all human breast cancers although it could be a useful marker for a subset of human breast tumours, what we will analyse in more detail in our upcoming studies.

Analysing the data from our TMA a positive significant association was found between overexpression of GLI1 and unfavourable overall survival outcome. This association has not been reported anywhere else so far, but similar tendencies were recently shown in human oesophageal cancer [36]. Patients with abundant GLI1 expression in the tumour (IRS > 6) had an estimated mean OS of 117 months (95% confidence interval [CI]: 105 to 125 months) compared to an estimated mean OS of 102 months (95% CI: 91 to 112 months) which was found in patients with lower levels of GLI1 expression (IRS = 6). Our data indicate that nuclear GLI1 overexpression being associated with unfavourable overall survival could be a phenomena found in a variety of human solid tumours that have been shown to express members of the Hh signalling pathway [2,5,6,8,37].

To assess the role of GLI1 for the progression of human breast cancer in more detail we next correlated GLI1 expression data to clinicopathological characteristics of the tumours. Interestingly we found a positive significant association between increased nuclear GLI1 expression and tumour stage, lymph node status of the analysed breast tumours supporting a role of GLI1 as a new prognostic marker. We also carried out multivariate analysis and found a significant association between advanced tumour stage (pT2 vs pT1) and increased likelihood of GLI1 overexpression in the analysed tumours (4.9 times; 95% CI 2.3 – 10.5). However associations between GLI1 overexpression and lymph node status as well as histological grade of the tumours as stated by univariate analysis were multivariate insignificant.

Similar to our study Kubo et al. (2004) correlated expression of GLI1 to clinicopathological characteristics of human breast tumours and found a significant association of GLI1 overexpression to oestrogen receptor status and histological subtype of the analysed tumours (*P *= 0.0216 and *P *= 0.0036) [35]. These correlations could not be stated through the results from our study and therefore we would not support the hypothesis of an involvement of the Hh pathway in the hormone-induced development of human breast carcinomas [35,38].

With lower abundance (median IRS = 6) GLI1 was also expressed in the nucleus of normal breast cells, and therefore we suggest a general involvement of GLI1 in transcriptional regulatory processes that are not only important within tumour development but also in normal breast cells. However Kubo et al. analysed normal breast epithelia (n = 52) and did not detect GLI1 expression in the nucleus of normal breast cells adjoining the analysed tumourous tissues.

So far the discrepancies between these two studies remain unsolved and should therefore be topic of further analyses characterizing the potential of GLI1 as a new potential prognostic marker of human breast cancer.

To find out more about the complexity of GLI1 signalling and its functional implications on human breast cancer a systematic characterisation of potential upstream and downstream targets of GLI1 in tumourous breast samples that are characterised by GLI1 overexpression would be an important approach. Interestingly the results from our statistical analyses showed a significant association between GLI1 overexpression and the presence of Hh member SHH in human breast tumours. Our observations indicate that not only GLI1 expression but more important its implication in the Hh signalling pathway might be a more precise characteristic of human breast cancers.

SHH and GLI are both members of the Hh signalling cascade which was first described in the fruit fly Drosophila [39] and it might be reasonable to assume that this signalling pathway is conserved and active in a proportion of human breast cancers. In mammals, a direct influence of SHH expression on increased GLI1 activity has been described in a variety of biological processes, e.g. during mouse limb bud development [40]. Furthermore increased expression of *SHH *mRNA in human colonic adenocarcinomas is known to correlate with downstream increased expression of GLI1 leading to promotion of cell proliferation [41]. In human cancer, sustained activity of Hh-GLI signalling is indicated to be essential in growth and survival of human prostate cancer cells [42]. The results from our correlation analyses between SHH and GLI1 are in accordance to these findings and may lead to the suggestion that GLI1 activity is mediated through the Hh initiating molecule SHH and that abundant levels of nuclear GLI1 expression observed in breast cancer could be a result of increased SHH activity. More interestingly Hh signalling is also active in human breast cancer stem cells and studies on *in vitro *culture systems showed that an activation of this pathway with Hh ligands promotes the self-renewal of mammary stem cells, and also increases proliferation of mammary progenitor cells as reflected by increased numbers of mammosphere-initiating cells and increased mammosphere size [43]. On regard of this an early oncogenic activation of Hh signalling as an initial step in breast cancer development starting at the level of tumour progenitor stem cells could be proposed.

To achieve therapeutic benefits of new oncogenic markers that are involved into tumour development, strategies have to be developed to inhibit their cancer initiating potential. Interestingly a number of molecules inhibiting the Hh signalling pathway have already been described. Cyclopamine for example is an inhibitory molecule for Hh signalling and has been shown to inhibit the proliferation of brain tumour cells which contain an active Hh signalling pathway [44]. GANT61 a hexahydropyrimidine derivative and GANT58 possessing a thiophene core with four pyridine rings, are two other small-molecule antagonists, which act in the nucleus to block GLI1 function and inhibit GLI1 mediated transcription [45]. Assuming that GLI1 might be involved into human breast cancer development and further proposing that GLI1 is activated through upstream members of the Hh signalling cascade, the mentioned Hh inhibitors could offer new drugs in the treatment of breast cancer as well.

In conclusion, we showed nuclear GLI1 overexpression in human breast carcinomas relative to normal breast tissue on the RNA and protein level. Interestingly, we also found a significant positive correlation between increased nuclear GLI1 expression and aggressive tumour characteristics (i.e. tumour stage and lymph node status). The aggressive behaviour of GLI1 positive breast cancers is supported by the finding that GLI1 overexpression is associated to unfavourable overall survival.

Our data raise the hypothesis that *GLI1 *represents a candidate oncogene in human breast cancer. Additional studies validating these data and further analysing possible interactions of GLI1 with other Hh signalling members are underway to clarify the role of this important developmental pathway for the pathogenesis of human breast cancer.

## Conclusion

GLI1 is an important transcriptional regulator within the Hh signalling cascade and may represent a novel biomarker in the development of human breast cancer with possible applications for future multimarker panels for early detection, molecular sub typing or personalised treatment. Increased levels of nuclear GLI1 expression in human tumourous breast tissues show a significant association towards lower overall survival outcome and a more aggressive tumour phenotype. Further studies will be conducted to validate overexpression of GLI1 in human breast tumours also on mRNA level and gain more insight into the relevance of Hh signalling for human breast cancer development.

## Abbreviations

Hh: Hedgehog; *GLI1*: Glioma-associated oncogene homolog 1; *SHH*: Sonic hedgehog homolog; *PTCH*: Patched homolog; *SMO*: Smoothened; *HER2*: Human epidermal growth factor receptor 2; *ERα*: Oestrogen receptor α; *PR*: Progesterone receptor; *GAPDH*: Glyeraldehyde-3-phosphate dehydrogenase; IHC: Immunohistochemistry; TMA: Tissue Microarray; OS: Overall survival; RFS: Recurrence-free survival; IRS: Immuno-Reactive-Score; H&E: Hematoxylin and eosin; DAB: 3,3'-Diaminobenzidine; ATCC: American Type Culture Collection; BCC: Basal cell carcinomas; CI: Confidence interval.

## Competing interests

The authors declare that they have no competing interests.

## Authors' contributions

AtH: designed the study, carried out the experiments, interpreted the data and wrote the manuscript; NB: analysed the immunohistochemical data and critically revised the manuscript; SvS and IL: supported with expertise in molecular biology techniques and in data interpretation; ECA: supported in the statistical data analysis; RK: participated in design and coordination of the study; ED conceived the study, participated in study design and coordination, molecular and data analysis, data interpretation and drafting of the manuscript.

## Pre-publication history

The pre-publication history for this paper can be accessed here:

http://www.biomedcentral.com/1471-2407/9/298/prepub
